# Parents’ Response to Children’s Performance and Children’s Self-Esteem: Parent–Child Relationship and Friendship Quality as Mediators

**DOI:** 10.3390/ijerph19106012

**Published:** 2022-05-15

**Authors:** Weina Li, Fenge Tan, Zongkui Zhou, Yukang Xue, Chuanhua Gu, Xizheng Xu

**Affiliations:** 1 Department of Management, Hunan Police Academy, Changsha 410138, China; liweina@hnpa.edu.cn (W.L.); xu_xizheng@126.com (X.X.); 2 Key Laboratory of Adolescent Cyberpsychology and Behavior, Central China Normal University, Ministry of Education, Wuhan 430079, China; tan672015428@mails.ccnu.edu.cn (F.T.); zhouzk@ccnu.edu.cn (Z.Z.); 3 Collaborative Innovation Center of Assessment toward Basic Education Quality, Central China Normal University Branch, Wuhan 430079, China; 4 Department of Educational and Counseling Psychology, University at Albany, State University of New York, Albany, NY 12222, USA; xueyukang@mails.ccnu.edu.cn

**Keywords:** children, parents’ response to children, parent–child relationship, friendship quality, self-esteem

## Abstract

Previous research has revealed that parents’ success-oriented response to children’s performance promotes children’s self-esteem, while failure-oriented response damages their self-esteem. However, the potential mediating mechanisms are unclear. Therefore, the present study investigated whether parent–child relationship and friendship quality mediated the relation between parents’ response to children’s performance and children’s self-esteem. For this purpose, 859 children in Central China completed the Parents’ Response to Children’s Performance Scale, Buchanan Scale of Closeness to Parents (CPS), Friendship Quality Questionnaire (simplified version), and Self-Perception Profile tests. Structural equation modeling (SEM) revealed that: (1) parents’ success-oriented response was positively associated with parent–child relationship, friendship quality, and children’s self-esteem. Parents’ failure-oriented response was negatively associated with parent–child relationship and children’s self-esteem, but it was positively associated with friendship quality. (2) Parent–child relationship and friendship quality were identified as the serial mediators between parents’ success- or failure-oriented response and children’s self-esteem. These findings suggest that parents’ failure-oriented response should be reduced and parents’ success-oriented response should be increased to develop children’s self-esteem and establish a sound social network system for children.

## 1. Introduction

Middle and late childhoods are the periods when various components of children’s self-concept coalesce to yield the general affective sense of self-worth referred to as self-esteem [1]. Self-esteem is crucial to children’s physical and mental health development. Children with high self-esteem are more likely to achieve academic success and be happy [2]. In addition, self-esteem is also a protective factor against various children’s psychological and behavioral problems [3]. The interaction between parents and children plays an essential role in children’s mental development [4]. Furthermore, previous research has shown that parents’ response to children’s performance plays a particular role in children’s psychological function [5]. Parents’ success-oriented response to children’s performance is positive to children’s self-esteem, while their failure-oriented response is related to children’s low self-esteem [6]. To date, few studies have examined the mediating mechanisms that “explain” how parents’ response to children’s performances is related to children’s self-esteem. However, the answer to this question is essential for maintaining and intervention of children’s self-esteem from the caregivers’ perspectives. Based on the theoretical model, this study will test the explanatory role of parent–child relationship and friend relationship quality in the relationship parents’ response and children’s self-esteem.

### 1.1. Parents’ Response to Children’s Performance and Children’s Self-Esteem

Parents often set goals for children according to social norms and cultural values when helping children adapt to school, society, and even more micro cultural value systems [7]. As the product of cultural values and socialization, those goals guide parenting practice. Research has found that parents’ different parenting goals for their children lead to different tendencies in success- or failure-response and eventually cause different influences on children’s mental development. Success-oriented parents are happy about their children’s good performance in study and still pay attention to the positive aspects of their performance (e.g., learning attitude, effort, and ability) when their performance is bad. However, failure-oriented parents pay more attention to negative aspects of their children’s performance no matter how they perform. For example, those parents may scold children simply because they did not achieve good marks.

Parents’ success-oriented responses suggest that they value developing a sense of self-worth [7]. According to social-cognitive developmental perspective theory, children may infer parents’ goals from their responses [8]. When parents use success-oriented responses more frequently, children assume that their parents care about their self-worth [9], which helps them focus on their self-esteem and feel appreciated and loved. According to self-determination theory [6], those feelings may satisfy children’s basic need for relatedness and love, promoting children’s essential psychological function development. Parents’ success-oriented response gives positive feedback on their children’s performance, and children will feel happier when they succeed [10], which boosts children’s self-esteem [11]. 

On the other hand, parents who value children’s efforts and self-improvement may be more likely to adopt failure-oriented responses [9], as these parents believe that adopting failure-oriented responses help their children realize what they need to improve and the importance of effort [7]. However, such responses may also pressure the children and make them think they can never do well enough, thereby increasing self-doubt and emotional distress [9] and decreasing self-esteem. The study found that failure-oriented responses may hurt children’s self-worth and inhibit their emotional function [12]. Additionally, some experimental and longitudinal studies have found that parents’ response to children’s performance predicts children’s self-esteem [6,9,10]. Therefore, we hypothesize that parents’ success-oriented response might positively predict children’s self-esteem, while parents’ failure-oriented response might negatively predict children’s self-esteem.

### 1.2. Mediating Role of Parent–Child Relationship and Friendship Quality

Studies have shown that parents’ success- or failure-oriented responses may affect children’s self-esteem, but the mechanism has not been wholly studied [6]. From the perspective of social networks, parent–child relationships and peer relationships are critical social-ecological sub-entities in children’s development [13]. A family is an essential place for children to obtain a sense of self-worth, and parent–child relationship quality is positively correlated with children’s self-esteem [14]. In addition, reciprocal friendship is also conducive to developing children’s self-esteem, and children with a higher level of friendship quality usually show a higher level of self-esteem [15]. Therefore, the mediating role of parent–child relationship and friendship quality in parents’ response to children’s performance and children’s self-esteem will be examined in our study.

#### 1.2.1. The Mediating Role of Parent–Child Relationship

Parenting involves parents’ response to children’s learning performance [7]. When parents adopt success-oriented responses, regardless of children’s performance, they give more positive feedback and praise to children’s performance, which is a crucial way of showing their warmth [16]. Parents’ warmth is an important component of the parent–child relationship [17]. In addition, children may actively construct their parents’ response to their performance in the view of social cognitive development theory [7]. Parents adopt success-oriented responses, conveying parents’ sincere concern for children’s self-value and caring for children’s well-being, which may cultivate a sense of connection between parents and children [6]. In addition, under the condition of success-oriented response, children may feel their parents’ acceptance of their behaviors and ideas as well as their parents’ trust and support, which are positive qualities of a parent–child relationship [17]. In addition, parenting creates an emotional atmosphere for establishing the parent–child relationship [18] and may affect the parent–child relationship [19]. Parents who adopt failure-oriented responses may be disappointed in their children, even blaming and criticizing them when they assess their children’s academic performance. [11]. Criticism and blame may arouse children’s negative emotions such as fear and anxiety, leading to distress or avoidance [20], which is not beneficial for establishing a good parent–child relationship. Therefore, we hypothesize that parents’ success-oriented response might positively predict the parent–child relationship, while parents’ failure-oriented response might negatively predict the parent–child relationship.

Family is an important source where children develop their perception of self-worth [21]. The parent–child relationship is dominant in developing individual social relations [22]. It has an essential impact on the development of children’s physical and mental health [23,24]. Schmidt and Padilla pointed out that when a person “experiences the love from another person, his self-esteem may be promoted” [25]. A close parent–child relationship means parents’ increased participation in children’s activities, more encouragement, support, and trust, and better communication, conveying information about their self-worth to children. When children think that parents can provide help and encouragement, it is helpful for children to cope with challenges, thereby contributing to overall greater self-esteem [14]. Moreover, Hong and Liu found that a good parent–child relationship positively affected children’s self-esteem [26]. Therefore, we hypothesize that the parent–child relationship might positively predict children’s self-esteem. Parenting style plays a vital role in establishing the parent–child relationship and eventually affects children’s psychological adaptability and well-being [27]. Parents’ response to children’s performance as a parenting style related to children’s academic performance affects the establishment of the parent–child relationship [7] and further affects the children’s self-esteem. Therefore, we hypothesize that the parent–child relationship is mediating between parents’ success- or failure-oriented response to children’s performance and self-esteem.

#### 1.2.2. The Mediating Role of Friendship Quality

In the view of the family systems theory [28], the family is the prominent place for individual socialization, especially in childhood. Social learning theory also provides theoretical support that parents’ response to children’s performance affects children’s friendship quality [24,29]. Children may observe and learn their parents’ responses to their performance and imitate them in a peer relationship. Parents who adopt success-oriented responses always pay attention to the positive aspects of children and convey encouragement and praise. Their children also show more affirmation and encouragement to their friends, which may enhance each other’s self-worth. Enhancing each other’s self-worth is an important feature of high friendship quality [30].

According to the social learning theory [31], children may imitate their parents’ behavioral responses according to parent–child interaction. Therefore, children may learn failure-oriented responses and pay more attention to the negative aspects of friends’ performance, making friends feel pressured and alienated and destroying friendship quality. In addition, parents’ failure-oriented response causes children to have more negative emotions about their poor performance [11]. Parents’ evaluation of children’s ability in a specific field is often related to children’s self-evaluation in this and other fields [32]. Consequently, parents’ failure-oriented response to children in school performance may cause the children to evaluate their academic ability and their social ability negatively. At the same time, the affirmation and recognition conveyed by parents’ success-oriented responses are not only conducive to the improvement of children’s social skills and interaction with peers. It can also reduce social anxiety and encourage children to actively confront peer harm [31], thereby increasing the positive factors of friendship quality and decreasing the negative ones. Therefore, we hypothesize that parents’ success-oriented responses might positively predict children’s friendship quality, while failure-oriented responses might negatively predict children’s friendship quality. 

The feeling of acceptance is one primary source of self-esteem. In addition, according to Harter’s symbolic interaction theory [33], children feel accepted when they have high-quality friendships (prosocial, intimacy, and loyalty) with their peers. Children who obtain high-quality friendships feel they are valuable [13]. They also have stronger subjective well-being and social adaptability, are more willing to cooperate with others and have a higher level of self-esteem [12]. In addition, longitudinal studies show that adolescents with friends have a higher self-esteem level in adulthood than adolescents without friends [34] and have fewer psychiatric symptoms. As friends are the primary source of social feedback for children, children with poor friendship quality may receive negative feedback [35], harming their self-esteem. On this basis, it can be concluded that children with a high level of friendship quality may have higher self-esteem [36].

Moreover, research indicated that friendship quality mediates the relationship between parenting (e.g., parental warmth) and children’s internalization and externalization [37]. Therefore, we hypothesize that friendship quality plays a mediating role between parents’ response to children’s performance and self-esteem.

### 1.3. Parent–Child Relationship and Friendship Quality as Sequential Mediators

From the social network perspective, both family and peer relations are within the microsystem that affects individual development [13]. Furthermore, those two micro-environments are interrelated. According to attachment theory, children’s perceived parent–child relationship impacts their perception of friendship quality [38] and further affects children’s development and adaptation. Therefore, parent–child relationship affects the quality of friendship.

To sum up, two styles of parental response to children have different characteristics and have different effects on the quality of parent–child relationships. It is predicted that success-oriented response positively impacts children’s perception of positive emotions in cognitive tasks [5]. Children enjoy time with their parents, which is conducive to developing positive parent–child relationship. In view of social learning theory, children in a good parent–child relationship are more likely to learn and imitate their parents’ social skills, which is beneficial for them to improve their interpersonal skills and emotional connection with friends [39]. Children may be supported and experience a sense of acceptance in close relationships with friends and parents, which is related to their sense of self-worth [13].

In the view of self-determination theory, children resist parents’ failure-oriented response [7], so parents’ failure-oriented response may intensify the contradiction between parents and children and then destroy the quality of the parent–child relationship. Moreover, the quality of children’s friendship partially depends on the quality of the parent–child relationship [40]. Friendship quality is positively correlated with children’s self-esteem [15]. In addition, studies have found that children’s social relations, except with their parents, play a mediating role between parent–child relationship and psychological adaptation [15,41,42]. Therefore, we hypothesize that parent–child relationship and friendship quality play a serial mediating role between the relationship of parents’ response to children’s performance and children’s self-esteem.

### 1.4. The Present Study

Although studies have tested the relationship between parents’ response to children’s performance and self-esteem, research gaps still exist. Studies on demonstrating the mediation effect of the parent–child relationship and friendship quality on the relationship between parents’ response to children’s performance and self-esteem are lacking. The answer to how parents’ responses impact children’s self-esteem is significant for maintaining and intervening in children’s self-esteem from the caregivers’ perspective. Thus, this study aimed to examine whether the parent–child relationship and friendship quality mediate the relationship between parents’ response to children’s performance and self-esteem in mainland China. The major hypotheses are as follows:

**Hypothesis** **1** **(H1).** *Parents’ success-oriented responses positively predict children’s self-esteem, while failure-oriented responses negatively predict children’s self-esteem.*

**Hypothesis** **2** **(H2).** *Parents’ success-oriented responses positively predict the parent–child relationship, while failure-oriented responses negatively predict parent–child relationships. The parent–child relationship positively predicts children’s self-esteem; the parent–child relationship plays a mediating role between parents’ response to children’s performance and children’s self-esteem.*

**Hypothesis** **3** **(H3).** *Parents’ success-oriented responses positively predict children’s friendship quality, while failure-oriented responses negatively predict children’s friendship quality. Friendship quality positively predicts children’s self-esteem; friendship quality plays a mediating role between parents’ response to children’s performance and children’s self-esteem.*

**Hypothesis** **4** **(H4).** *Parent–child relationship and friendship quality play a serial mediating role in the relationship of parents’ response to children’s performance and children’s self-esteem.*

## 2. Materials and Methods

### 2.1. Participants and Procedure

Random cluster sampling was conducted to recruit participants from grade 3 to grade 6 in three public primary schools in Central China. They are all from middle-sized cities with middle-income households. We distributed 952 questionnaires. After excluding questionnaires with problematic data (e.g., incomplete or consistent answers), 859 valid questionnaires were left. The recovery rate of the questionnaire was 90.23%. The participants’ age ranged from 9 to 14 years (M = 11.37, SD = 1.29), including 467 boys (54.40%). The number of participants from grade 3 to grade 6 was 243, 205, 174, and 257, respectively. The Research Ethics Committee of the university with which the first author was affiliated approved the study.

Ethical procedures were followed throughout the study. Firstly, the first author’s university and the Department of Education approved the study before data collection. Secondly, the consent of the leaders of the participating schools, teachers, students, and their parents was obtained before data collection. Thirdly, taking the class as a unit, the group sampling method was used for collective measurement. The researchers are graduate students majoring in psychology and have undergone professional testing training. The researchers gave unified instructions. After confirming that the students understood the questionnaire’s instructions, the researchers asked them to start answering independently. Subsequently, all questionnaires were collected on the spot. After the test, the researchers explained the purpose of the study to the students and gave them gifts in return.

### 2.2. Measures

#### 2.2.1. Parents’ Response to Children’s Performance

The scale of the Chinese version on Parents’ Response to Children’s Performance was used in this study [11]. The scale contains 16 items that could be classified into two subscales: success-oriented response scale and failure-oriented response scale. The two dimensions have 8 items apiece. The students were asked to select the option based on their recall that best describes their parents’ expressions after scoring either a very good or a very poor grade in one test. Examples of items on the success-oriented scale are: “My parents may be excited because of my good results when I do a good job,” “My parents may pay attention to what I answered correctly.” On the failure-oriented scale: “My parent may ask why I did not get a full score when I get a good result in the test,” “My parents may be very disappointed with me w hen I get a poor result.” The scale was scored by a five-point Likert scale ranging from 1 to 5, with 1 for “never” and 5 for “always”. Higher total scores indicated more obvious the parental responses. The scale was a useful tool with satisfactory reliability and validity among the Chinese subjects [6]. This study’s Cronbach alpha coefficients for the two subscales were 0.76 and 0.80, respectively.

#### 2.2.2. Parent–Child Relationship

The Closeness to Parents Scale (CPS) was developed by Buchanan et al. [43]. According to the actual situation, children score their father–child and mother–child relationship on nine items each (e.g., “How close do you feel to your father/mother?”). The mean value of the two is used as the score of parent–child relationship quality. Items are rated from 1 to 5, 1 for “not at all true” and 5 for “completely true”. The higher the score, the better the parent–child relationship. The scale has been widely used in Chinese children, with good reliability and validity in previous studies [44,45,46,47]. In this study, Cronbach’s alpha coefficients were 0.89 and 0.89 for the two sub-scales, respectively.

#### 2.2.3. Friendship Quality

Children’s friendship quality was assessed through a simplified version of the Friendship Quality Questionnaire (FQQ) designed by Parker and Asher [48] and prepared by peer nomination [13]. Three items with the highest load among the six dimensions of the original scale (affirmation and care, help and guidance, company and entertainment, intimate exposure and communication, conflict resolution strategies, conflict, and betrayal) were selected to form a simplified version of the questionnaire involving 18 items. The participants were asked to evaluate the friendship quality with their best friends (e.g., “We always sit together whenever we have the opportunity”). A five-point Likert scale scored the scale, 1 for “not at all true” and 5 for “completely true”. Three items on conflict and betrayal were scored reversely and were added with the scores of other items. Higher scores indicated better friendship quality. This scale was initially designed for children and has been widely used among Chinese primary school students, and it has good reliability and validity [49,50]. In this study, the Cronbach alpha for this questionnaire was 0.87.

#### 2.2.4. Self-Esteem

Self-Perception Profile for Children (SPPC) developed by Harter was used in this study [51]. The scale has been used for 8-year-old children and older [52]. The scale includes 6 dimensions and 36 items, including general self-perception, academic self-perception, social self-perception, sports self-perception, physical self-perception, and behavioral self-perception. The six items from the general self-perception dimension were used to measure individual overall self-esteem in this study (e.g., “Some children are often dissatisfied with themselves, while others are very satisfied with themselves.”). Students were invited to rank their responses on a four point-Likert scale. The scores of three items were reversed for analysis. Higher total scores indicate higher overall self-esteem. The scale has been widely used in China with good reliability and validity [53,54]. In this study, the Cronbach alpha was 0.69.

### 2.3. Data Analysis

IBM SPSS 26.0 was used for the common method deviation test and descriptive statistical analysis, and Mplus 8.3 was used for SEM to test the mediating effect [55]. Full information maximum likelihood (FIML) estimation was used to process missing data [56]. The average score of all scales was used in the relevant analysis, and each variable was treated as a latent variable when testing the mediating effect. As in Erceg-Hurn and Mirosevich’s study [57], 5000 samples were sampled using the deviation corrected percentile Bootstrap method to test the mediating effect.

## 3. Results

### 3.1. Common Method Bias Test

To control the method biases in this study, we have employed several measures in developing the instrument, such as explaining the anonymous filling in the test guidance, keeping all data strictly confidential and only for scientific research, and setting reverse scoring questions. In addition, we also adopted the common method bias testing for statistical remedies. Harman’s single-factor test was conducted [58]. Based on the analysis, 14 factors had eigenvalues greater than one and could jointly explain 18.76% of the variance, which was less than 40%. Therefore, there was no critical common method bias in this study.

### 3.2. Preliminary Analyses

Means and standard deviations of the study variables were calculated. As shown in Table 1, success-oriented response was positively correlated with parent–child relationship (*p* < 0.01), friendship quality (*p* < 0.01), and self-esteem (*p* < 0.01). Failure-oriented response was negatively correlated with parent–child relationship (*p* < 0.01) and self-esteem (*p* < 0.01). However, the failure-oriented response was positively correlated with friendship quality (*p* < 0.05). Parent–child relationship was positively correlated with friendship quality (*p* < 0.01) and self-esteem (*p* < 0.01). Friendship quality was positively correlated with self-esteem (*p* < 0.01). Gender was negatively correlated with friendship quality (*p* < 0.01). Grade was positively correlated with failure-oriented response (*p* < 0.05), and friendship quality (*p* < 0.01) was negatively correlated with parent–child relationship (*p* < 0.01). Therefore, gender and grade were included in the model as control variables.

### 3.3. Mediation Model with Parent–Child Relationship and Friendship Quality

#### 3.3.1. Test of Measurement Model

Results demonstrated that the measurement model has a good fit index (*χ^2^/d_f_* = 2.73, *RMSEA* = 0.05, *CFI* = 0.96, *TLI* = 0.96, and *SRMR* = 0.04). Results also show that all the observed variables had significant factor loadings on the latent variable. The standard factor loadings of all the observed variables were between 0.62 and 0.80, which are all acceptable.

**Figure 1:**
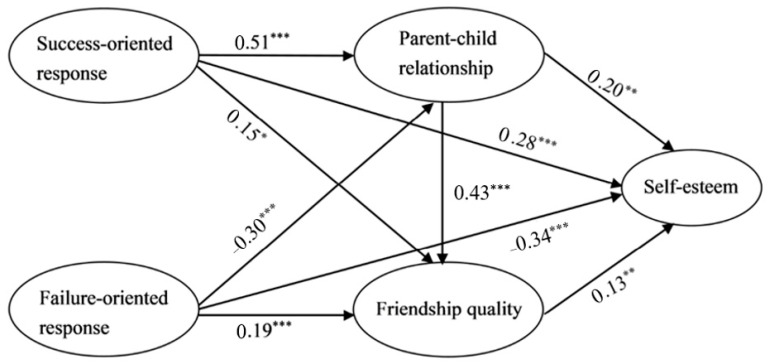
Serial mediating role of parent–child relationship and friendship quality (*N* = 859). * *p* < 0.05, ** *p* < 0.01, *** *p* < 0.001.

The predictive effect of two different parents’ responses to their children’s performance on children’s self-esteem was tested before testing the model with mediating variables in this study. The results of the SEM show a high fitting degree. (*χ^2^/d_f_* = 5.14, *RMSEA* = 0.07, *CFI* = 0.95, *TLI* = 0.92, and *SRMR* = 0.05). First, the regression results of the total sample confirmed that parents’ success-oriented response positively predicted children’s self-esteem (*β* = 0.43, *p* < 0.001), and parents’ failure-oriented response negatively predicted children’s self-esteem (*β* = −0.39, *p* < 0.001). In addition, with participants’ gender and grade as control variables, the serial mediating model involving the parent–child relationship and friendship quality was tested. The results showed that the fitting of the SEM reached a high level (*χ^2^/d_f_* = 2.91, *RMSEA* = 0.05, *CFI* = 0.95, *TLI* = 0.94, and *SRMR* = 0.05). All paths reached a significant level (as shown in Figure 1).

The results confirmed that: (1) parents’ success-oriented response positively predicted parent–child relationship (*β* = 0.51, *p* < 0.001), friendship quality (*β* = 0.15, *p* < 0.05), and self-esteem (*β* = 0.28, *p* < 0.001). (2) Parents’ failure-oriented response negatively predicted parent–child relationship (*β* = −0.30, *p* < 0.001) and self-esteem (*β* = −0.34, *p* < 0.001), while positively predicting friendship quality (*β* = 0.19, *p* < 0.001). (3) Parent–child relationship positively predicted friendship quality (*β* = 0.43, *p* < 0.001) and self-esteem (*β* = 0.20, *p* < 0.01). (4) Friendship quality positively predicted self-esteem (*β* = 0.13, *p* < 0.01).

#### 3.3.2. Significance Test of Mediating Effect

Lastly, we examined the direct and indirect effects of parents’ success- or failure-oriented response on children’s self-esteem. As shown in Table 2, the results for the whole sample indicated that: (1) parents’ success- or failure-oriented response to children’s performance had a significant direct influence on children’s self-esteem; (2) the indirect effect of Success-oriented response →Parent–child relationship → Self-esteem was significant (*β* = 0.10, 95% CI ranged from 0.04 to 0.17); (3) Success-oriented response →Friendship quality →Self-esteem was not significant (*β* = 0.02, 95% CI ranged from −0.002 to 0.04); (4) the indirect effect of Success-oriented response →Parent–child relationship →Friendship quality→ Self-esteem was significant (*β* = 0.03, 95% CI ranged from 0.01 to 0.05), and the total indirect effect value of parent–child relationship and friendship quality was 0.148, accounting for 34.58% of the total effect; (5) the indirect effect of Failure-oriented response→ Parent–child relationship →Self-esteem was significant (*β* = −0.06, 95% CI ranged from −0.10 to −0.02); (6) the indirect effect of Failure-oriented response →Friendship quality →Self-esteem was significant (*β* = 0.03, 95% CI ranged from 0.0001 to 0.05); and (7) the indirect effect of Failure-oriented response →Parent–child relationship →Friendship quality →Self-esteem was significant (*β* = −0.02, 95% CI ranged from −0.03 to −0.002), and the total indirect effect value of parent–child relationship and friendship quality was −0.052, accounting for 13.37% of the total effect. All these findings jointly support the serial mediation model for the total sample, which means that parents’ success- or failure-oriented response indirectly impacted children’s self-esteem through parent–child relationship and friendship quality in turn.

## 4. Discussion

Built on the characteristics of parents’ response to their children’s performance, views of the social network ecosystem, self-determination theory, and social learning theory, the study examined the mediating role of the parent–child relationship and friendship quality between parents’ responses and children’s self-esteem. The study found that parents’ success- or failure-oriented responses directly predicted children’s self-esteem. Moreover, parents’ success- or failure-oriented responses also indirectly predicted children’s self-esteem through the parent–child relationship and friendship quality.

### 4.1. Parents’ Response to Children’s Performance and Children’s Self-Esteem

This study found that parents’ response to children’s performance directly predicted children’s self-esteem. Specifically, success-oriented responses positively predicted children’s self-esteem, while failure-oriented responses negatively predicted children’s self-esteem. These results confirmed Hypothesis 1 and were consistent with previous studies [6]. When parents adopt success-oriented responses, parents’ praise and recognition may make children feel superior and valued; they foster a sense of connection in their children. According to the self-determination theory [59,60], this sense of connection can meet children’s needs for relatedness to improve their self-esteem [6]. In addition, after interacting with parents who adopt success-oriented responses, the children are more positive in their abilities to complete cognitive tasks and experience more active emotions [9], which promotes the development of children’s self-esteem.

However, the failure-oriented responses make children feel pressured to meet their parents’ growing expectations. Therefore, their autonomy and competence needs are not met, so the development of children’s self-esteem is inhibited [6]. In addition, the greater the frequency of parents’ failure-oriented responses, the greater the sensitivity of children to failure and the lower their sensitivity to success [11]. Children experience more frustration for failure and less happiness for success, which magnifies the threat of failure to self-esteem and reduces the promoting effect of success on protecting self-esteem from risk factors. Thus, it is understandable that parents’ success-oriented responses enhance children’s self-esteem, while parents’ failure-oriented responses reduce children’s self-esteem.

### 4.2. The Mediating Role of Parent–Child Relationship

This study has confirmed the mediating role of the parent–child relationship in the path from parents’ response to children’s performance to children’s self-esteem, consistent with the view of family system theory [28]. Parents’ success-oriented response conveys positive emotions [11] and induces children to produce more positive emotions, conducive to establishing a good parent–child relationship with positive emotions [12]. Parents’ success-oriented response permeates parent–child interaction in multiple fields. It strengthens children’s experience of self-determination, satisfaction, and connection with parents, which increases factors to construct the parent–child relationship except for learning activities. The parent–child relationship could positively predict children’s self-esteem. This finding is consistent with previous research results [14]. With a high-quality parent–child relationship, children believe they can receive more support and encouragement from their parents, which is conducive to developing children’s self-esteem [61]. Therefore, the findings suggested that the parent–child relationship played a significant mediating role between parents’ success-oriented response and children’s self-esteem.

In the interaction with children, parents’ failure-oriented response conveys more negative statements to their children [12], such as anger or blame [11], which may aggravate children’s distress after failure. Failure itself is a threat to the children with parents’ negative statements, and other behaviors cannot alleviate children’s distress and may convey to children that parents do not care about their feelings, which may damage the parent–child relationship. The damage to parent–child relationship makes the children feel that they do not receive the care and love from their parents and lack a sense of belonging, which does not help develop the children’s self-esteem [26]. The decline in the parent–child relationship level means that the support from parents is reduced. In fact, the impact of parental support on children’s self-esteem is more obvious than other sources of support [14]. Therefore, this study has provided evidence to support that parents’ failure-oriented response may lead to a decline in the parent–child relationship level, which has an adverse impact on children’s self-esteem.

### 4.3. The Mediating Role of Friendship Quality

Inconsistent with part of Hypothesis 3, the mediating effect of friendship quality between parents’ success-oriented response and children’s self-esteem was not significant. It is possible that success-oriented responses may convey positive concern [11] and promote the establishment of the parent–child relationship; however, due to their psychological development level limitations, this cannot necessarily lead children to perceive the success-oriented response as an interpersonal skill to learn.As a result, success-oriented response positively predicts friendship quality, but the effect is not obvious. Furthermore, parents lead the role of the primary mentor in children’s emotional, social, and cognitive development [29]. Only individuals in adolescence can gradually transfer interpersonal communication to members outside the family. Additionally, eventually, obtaining close friendships and promoting their own emotional and social ability and autonomy development become their essential development task [62]. Therefore, children are less likely to link friendship quality to self-esteem than teenagers. Therefore, it is understandable that the mediating effect of friendship quality between success-oriented responses and children’s self-esteem was not significant.

The study found that friendship quality is mediating between parents’ failure-oriented response and children’s self-esteem. However, parents’ failure-oriented response positively predicts children’s friendship quality, inconsistent with Hypothesis 3. School is an important environment for children, and failure is an essential threat to children due to failure’s practical and potential consequences [7]. Therefore, children may require interpersonal support to deal with this threat. However, parents’ failure-oriented response will convey negative emotions such as disappointment [1] and lack of sensitivity to children’s needs of relatedness [24]. Therefore, children may seek support from friendship to compensate for parental inadequacies in providing interpersonal support [63]. Children may reject parents’ failure-oriented response [12]. Therefore, too many parents’ failure-oriented responses may turn the family into a place where children do not wish to remain and push them into friendship [64]. In addition, according to the social learning theory [24], children may learn and internalize their parents’ failure-oriented response and then develop a similar response to their own performance, demonstrating more humility and facilitating academic performance [6]. This behavior is consistent with traditional Chinese culture [11], thereby increasing children’s popularity in interpersonal interactions. Therefore, there is a significant positive correlation between parents’ failure-oriented response and children’s friendship quality. In a high-quality friendship, children feel more warmth, which is related to children’s high level of self-esteem, consistent with previous research results [34]. Therefore, this study has provided evidence to support the mediating role of friendship quality in failure-oriented responses and children’s self-esteem.

### 4.4. The Serial Mediation Role of Parent–Child Relationship and Friendship Quality

Parents’ success-oriented response means emphasizing children’s good performance and giving them positive responses to meet children’s needs for positive self-care [11], which may create an atmosphere for establishing a good parent–child relationship. A high-quality parent–child relationship helps parents guide children to get on with some friends and stay away from negative peers to help children develop a normal friendships [65]. Children with high-quality parent–child relationships are more likely to identify with their parents’ values and norms, which helps alleviate conflicts in friendship [39]. Positive friendship quality helps children develop a high level of self-esteem [66]. Which is consistent with the view of social support theory. The theory holds that high-quality friendship can provide children with support, including emotion, social skills, and how to value and respect others, which may promote children’s positive self-adjustment [35]. Therefore, this study found that parents’ success-oriented response impacts children’s self-esteem via the serial mediation of parent–child relationship and friendship quality.

Facing academic failure, children need more of their parents’ care and encouragement. However, if parents adopt a failure-oriented response to pay more attention to children’s failure performance, and express negative emotions such as disappointment at that time [11], they are not sensitive to children’s needs [7], which may lead to an increase in the sense of distance between children and their parents and a destroyed parent–child relationship. A poor parent–child relationship may hinder children from internalizing their parents’ values and norms, which can help children alleviate conflicts in friendship and help children develop a positive friendship [39]. Therefore, the parent–child relationship affects children’s other social relations, and bad parent–child relationships destroy the quality of friendship, which is consistent with attachment theory. Negative friendship quality is related to children’s perception of low social acceptance [35], and the perception of non-acceptance may make children doubt their self-worth and reduce their self-esteem. Therefore, this study found that parents’ failure-oriented response impacted children’s self-esteem via the serial mediation of parent–child relationship and friendship quality.

## 5. Conclusions, Limitations, and Implications

As the empirical exploration of the potential mechanism of parents’ response to children’s performance on children’s self-esteem in China, this study has very preliminary but noticeable findings. First, parents’ success-oriented response has a direct positive impact on children’s self-esteem, while parents’ failure-oriented response is negative to children’s self-esteem. Second, parents’ success- or failure-oriented response affects children’s self-esteem through parent-child relationships as a mediating factor. Parents’ failure-oriented responses affect children’s self-esteem by mediating children’s friendship quality. Third, parent-child relationship and friendship quality were identified as the serial mediators between parents’ response to children’s performance and children’s self-esteem.

This study, however, has three major limitations. First, we collected self-report data, which should be interpreted cautiously due to social desirability and recall biases. Further studies with integrated methods (i.e., teacher-, self-, and peer- report) are needed. Second, the children in this study are all from middle-sized urban in China. Thus, the representativeness of the subject is limited, and results should be treated with caution for generalization. Third, it is impossible to establish a causal relationship between these variables because this is a cross-sectional study with exploratory results [31]. Nonetheless, robust theoretical and empirical research supports the demonstration of causality between variables [6,9,10,38]. Future research can design supplemental experiments or longitudinal studies to investigate causality based on this study.

Despite these limitations, these findings have significant implications for maintaining and fostering children’s self-esteem. First, consistent with previous studies [31], our findings suggest that a success-oriented response is a significant promotional factor for children’s self-esteem. Thus, encouraging parents to adopt more success-oriented responses than failure-oriented responses should be the focus of interventions aimed at raising children’s self-esteem. Second, given that the parent–child relationship is an important mechanism through which parents’ response impacts children’s self-esteem, accurately screening the parent–child relationship and training parents with the essential skills to improve the parent–child relationship should be the primary goal in programs aiming to maintain children’s self-esteem. Third, as friendship quality also mediates the relationship between parents’ response and children’s self-esteem, caregivers paying attention to optimizing friendship quality may help maintain children’s self-esteem. Finally, our results show multiple mediators linking parents’ response to children’s self-esteem. Interventions that target the two mediators at the same time are more likely to be useful than targeting any single mediator. Additionally, programs that aim to improve the parent–child relationships and friendship quality should be accompanied by a complementary focus on training parents to consciously adopt success-oriented responses. This is vital because the relationship between parents’ response and children’s self-esteem is only partially mediated by the two mediators, and failure-oriented response is a risk factor that damages parent-child relationships and children’s self-esteem.

## Figures and Tables

**Table 1:** Means, standard deviations, and correlation matrix (*N* = 859).

Variables	*M*	*SD*	1	2	3	4	5	6	7
1 Gender	0.54	0.50	1						
2 Grade	4.54	1.16	0.04	1					
2 Success-oriented response	2.99	0.95	0.02	−0.04	1				
3 Failure-oriented response	2.57	0.95	0.06	0.07 *	0.07	1			
4 Parent–child relationship	3.34	0.96	−0.03	−0.11 **	0.37 **	−0.22 **	1		
5 Friendship quality	3.72	0.79	−0.16 **	0.12 **	0.29 **	0.08 *	0.34 **	1	
6 Self-esteem	2.73	0.62	0.06	−0.02	0.30 **	−0.28 **	0.35 **	0.21 **	1

Note: Gender is a dummy variable, male = 0, female = 1, the mean represents the proportion of girls. * *p* < 0.05, ** *p* < 0.01.

**Table 2:** Indirect effects of parents’ success- or failure-oriented response on children’s self-esteem.

	Indirect Effect Values	*SE*	95% Confidence Interval
Total indirect effects of SOR	0.148	0.031	[0.086, 0.211]
SOR→PCR→SEM	0.101	0.032	[0.038, 0.165]
SOR→FQ→SEM	0.019	0.010	[−0.002, 0.039]
SOR→PCR→FQ→SEM	0.028	0.012	[0.005, 0.051]
Total indirect effects of FOR	−0.052	0.024	[−0.098, −0.006]
FOR→PCR→SEM	−0.060	0.021	[−0.100, −0.019]
FOR→FQ→SEM	0.025	0.012	[0.001, 0.048]
FOR→PCR→FQ→SEM	−0.017	0.008	[−0.031, −0.002]

Note: SOR = success-oriented responses; FOR = failure-oriented responses; PCR = parent–child relationship; FQ = friendship quality; and SEM = self-esteem.

## Data Availability

The datasets analyzed during the current study are available from the corresponding author upon reasonable request.
